# Spatial and temporal country-wide survey of temephos resistance in Brazilian populations of *Aedes aegypti*

**DOI:** 10.1590/0074-02760150409

**Published:** 2016-05

**Authors:** Mateus Chediak, Fabiano G Pimenta, Giovanini E Coelho, Ima A Braga, José Bento P Lima, Karina Ribeiro LJ Cavalcante, Lindemberg C de Sousa, Maria Alice V de Melo-Santos, Maria de Lourdes da G Macoris, Ana Paula de Araújo, Constância Flávia J Ayres, Maria Teresa M Andrighetti, Ricristhi Gonçalves de A Gomes, Kauara B Campos, Raul Narciso C Guedes

**Affiliations:** 1 Universidade Federal de Viçosa, Departamento de Entomologia, Viçosa, MG, Brasil; 2 Secretaria Municipal de Saúde de Belo Horizonte, Belo Horizonte, MG, Brasil; 3 Ministério da Saúde, Secretaria de Vigilância em Saúde, Coordenação Geral do Programa Nacional de Controle da Dengue, Brasília, DF, Brasil; 4 Secretaria Municipal de Saúde de São Domingos do Prata, São Domingos do Prata, MG, Brasil; 5 Fundação Oswaldo Cruz, Instituto Oswaldo Cruz, Laboratório de Fisiologia e Controle de Artrópodes Vetores, Rio de Janeiro, RJ, Brasil; 6 Ministério da Saúde, Secretaria de Vigilância em Saúde, Coordenação Geral de Laboratórios de Saúde Pública, Brasília, DF, Brasil; 7 Secretaria de Saúde do Ceará, Núcleo de Controle de Vetores, Laboratório de Entomologia, Fortaleza, CE, Brasil; 8 Fundação Oswaldo Cruz, Centro de Pesquisas Aggeu Magalhães, Recife, PE, Brasil; 9 Secretaria de Saúde de São Paulo, Superintendência de Controle de Endemias, Marília, SP, Brasil

**Keywords:** insecticide resistance survey, dengue, distance interpolation, distribution maps, mosquito larvae

## Abstract

The organophosphate temephos has been the main insecticide used against larvae of the dengue and yellow fever mosquito (*Aedes aegypti*) in Brazil since the mid-1980s. Reports of resistance date back to 1995; however, no systematic reports of widespread temephos resistance have occurred to date. As resistance investigation is paramount for strategic decision-making by health officials, our objective here was to investigate the spatial and temporal spread of temephos resistance in *Ae. aegypti* in Brazil for the last 12 years using discriminating temephos concentrations and the bioassay protocols of the World Health Organization. The mortality results obtained were subjected to spatial analysis for distance interpolation using semi-variance models to generate maps that depict the spread of temephos resistance in Brazil since 1999. The problem has been expanding. Since 2002-2003, approximately half the country has exhibited mosquito populations resistant to temephos. The frequency of temephos resistance and, likely, control failures, which start when the insecticide mortality level drops below 80%, has increased even further since 2004. Few parts of Brazil are able to achieve the target 80% efficacy threshold by 2010/2011, resulting in a significant risk of control failure by temephos in most of the country. The widespread resistance to temephos in Brazilian *Ae. aegypti* populations greatly compromise effective mosquito control efforts using this insecticide and indicates the urgent need to identify alternative insecticides aided by the preventive elimination of potential mosquito breeding sites.

Vector-borne neglected (tropical) diseases such as dengue are an increasing worldwide issue of concern, particularly given current rates of urbanisation, international travel and trade, and climate change, all of which favor the spread of such diseases and their vectors (Hsieh & Chen 2009, Guzman et al. 2010, Gubler 2012). Mass gatherings and large sporting events are also associated with higher risks of health incidents. The 2014 FIFA World Cup held in Brazil is an example that drew attention and incited debate that focused particularly on dengue due to potential vector outbreaks (Hay 2013). The concern is understandable and justifiable, even if the risks were generally small (Lowe et al. 2014, van Panhuis et al. 2014). As a result, no serious incident came to past. The 2016 Olympic Games to be held in Rio de Janeiro are bound to draw a similar level of international attention.

The lack of effective vaccines or pharmaceutical treatments for dengue, typical of the neglected diseases, places mosquito vector control in the forefront of prevention efforts for this disease (Gubler 2004, Halstead 2012). This scenario prevails throughout the affected tropical and subtropical regions of the world, which roughly encompasses about half of the global population (Guzman et al. 2010). Control of the dengue mosquito vector [*Aedes aegypti* (L.)], which also transmits chikungunya, zika and yellow fever (YF) (thus the common name “yellow fever mosquito”), relies heavily on insecticide use - but there are few compounds available and their use is usually guided by the countries’ health officials (OPAS 1995, Funasa 2001, 2002, Braga & Valle 2007, Araújo et al. 2013, Macoris et al. 2014, Tomé et al. 2014).

The organophosphate temephos is globally the most commonly used insecticide against mosquito larvae due to its high efficacy, low cost and low vertebrate toxicity (WHO 2009). The result of this overreliance on temephos in controlling YF mosquito larvae is evolution and spread of temephos resistance among populations of this pest species. Such resistance has been detected in various countries since 1995 (Macoris et al. 1995, Mazzari & Georghiou 1995, Rawlins & Wan 1995, Bisset Lazcano et al. 2009, Melo-Santos et al. 2010, Bisset et al. 2013, Grisales et al. 2013). Furthermore, the use of temephos for the control of larvae of *Ae. aegypti* also apparently led to incidental selection for temephos resistance in co-occurring mosquito species populations (Campos & Andrade 2003, Alves et al. 2011, Phophiro et al. 2011, Amorim et al. 2013), as has also been reported among other co-occurring arthropod pest species (Guedes et al. 2016).

Routine applications of temephos against mosquito larvae in Brazil began in the 1980s (Funasa 1994, 2001, Sucen 1997). The initial suppression of *Ae. aegypti* in Brazil by 1955 was followed by its subsequent return in the 1970s (Schatzmayr 2000, Lourenço-de-Oliveira et al. 2004). Dengue became endemic in the country and has become an increasingly serious problem since 1986 despite established vector control programs in the country that still continue today (Lourenço-de-Oliveira et al. 2004, Maciel-de-Freitas et al. 2014). By the 1990s, concern emerged in Brazil regarding likely control failures and detection of temephos-resistant mosquito populations, which led to systematic surveys of insecticide resistance in the country and a series of reports on the phenomenon (Macoris et al. 1995, 2003, 2007, 2014, Campos & Andrade 2003, Lima et al. 2003, 2006, Melo-Santos et al. 2010, Gambarra et al. 2013, Diniz et al. 2014).

A few studies on the underlying mechanisms of temephos resistance followed the initial detection of this phenomenon in Brazil. Despite of an initial report of altered (acetylcholinesterase) target site sensitivity detected in a Brazilian population of *Ae. aegypti* resistant to temephos from Uberlândia (MG), current evidence suggests the prevalence of enhanced detoxification by metabolising enzymes in an apparently mixed pattern (Braga & Valle 2007, Melo-Santos et al. 2010, Lima et al. 2011, Gambarra et al. 2013). Congruent findings have been reported from other countries as well (Bisset Lazcano et al. 2009, Bisset et al. 2013, Grisales et al. 2013). Furthermore, recent transcriptome (i.e., the set of all mRNA molecules from a cell) evidence indicates upregulation of detoxification enzymes in insecticide-resistant mosquitoes (Reyes-Solis et al. 2014, Saavedra-Rodriguez et al. 2014). These findings reinforce the perception that multiple metabolic genes are involved in temephos resistance in *Ae. aegypti*, but with the prevalence of esterase rather than glutathione*-S-*transferase gene expression (Reyes-Solis et al. 2014, Saavedra-Rodriguez et al. 2014).

Temephos resistance monitoring in populations of the YF mosquito were underway in Brazil by the late 1990s in response to the increasing incidence of dengue in the country (Braga & Valle 2007). Scientific reports of the incidence of temephos resistance have increased since then (Campos & Andrade 2003, Lima et al. 2003, 2006, Macoris et al. 2003, 2007, Melo-Santos et al. 2010, Gambarra et al. 2013, Diniz et al. 2014), but no comprehensive dataset is currently available and no area-wide description of the phenomenon of temephos resistance and its spread has been attempted despite the strategic importance of such information in guiding control policies, protocols and decision-making by Brazilian health officials. The current effort took advantage of the dataset gathered by the National Network of Insecticide Resistance Monitoring (MoReNAa) in *Ae. aegypti* under the tutelage of the National Program of Dengue Control from the Office of Health Surveillance of the Brazilian Ministry of Health (Brasília, DF, Brazil). The objective of our study was to recognise the spatial and temporal spread of temephos resistance in Brazil for the past 12 years, which we hypothesized, has been acute and has likely encompassed the entire country since 2010.

Our spatial and temporal survey of temephos resistance was performed using standardised procedures for insect sampling and temephos bioassays from the WHO (1981) that were countersigned by the laboratories involved (from MoReNAa) with the support of the Centers for Disease Control and Prevention (CCD, USA), Pan-American Health Organization and the World Health Organization (Braga et al. 2004, Macoris et al. 2005, Braga & Valle 2007). The data obtained was subjected to kriging to select suitable semivariogram models for distance interpolation with the goal of generating geospatial maps of the frequency of temephos resistance in Brazilian populations of *Ae. aegypti*.

## MATERIALS AND METHODS

**Fig. 1:**
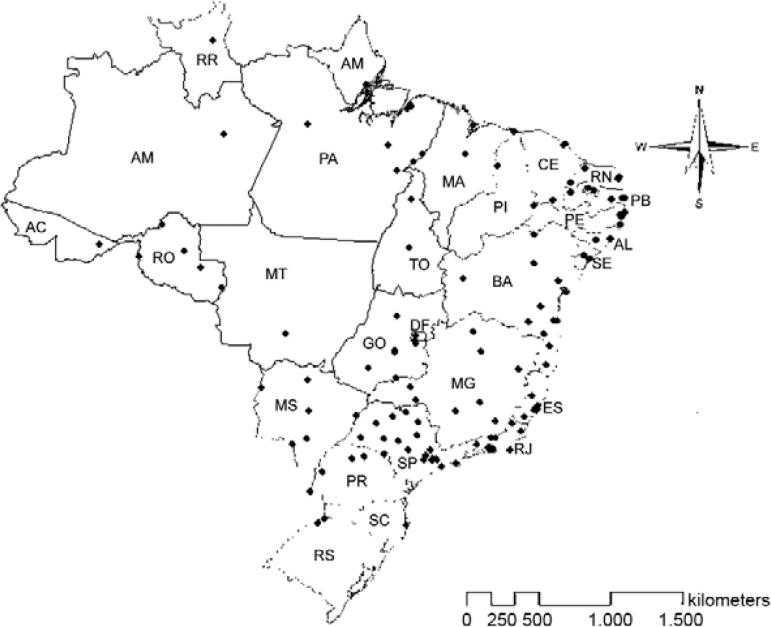
: distribution of the sampling sites of the populations of the yellow fever mosquito *Aedes aegypti* used in the spatio-temporal survey of temephos resistance in Brazil. Identification for each sampling site and its coordinates are listed in Table I.

*Insects and insecticide* - Mosquito populations were sampled through the MoReNAa (Table I, Fig. 1) as described by Macoris et al. (2003). Briefly, between 100-200 oviposition traps (i.e., ovitraps) were used for this purpose in each city. The ovitraps were placed outdoors in a grid pattern for four weeks, always in the second semester of each year (Fay & Eliason 1966, Jakob & Bevier 1969, Funasa 1999). Egg clutches thus collected were used to establish laboratory colonies of over 3,000 individuals from each city (i.e., sampling site). First-generation larvae raised in the laboratory were used in the bioassays (Lima et al. 2003, Macoris et al. 2003). Technical grade temephos (> 90% pure) was obtained from the Brazilian Ministry of Health and diluted with acetone at the desired concentration for subsequent use in the diagnostic bioassays.

**TABLE I:** Sample site identification and geographical coordinates of collection sites for populations of the yellow fever mosquito *Aedes aegypti* used in the spatio-temporal survey of temephos resistance in Brazil

Region	State	City	Longitude	Latitude
North	Rondônia (RO)	Cacoal	-61,447222	-11,438611
North	Rondônia (RO)	Guajará-Mirim	-65,339444	-10,782778
North	Rondônia (RO)	Porto Velho	-63,903889	-8,761944
North	Rondônia (RO)	Jaru	-62,466389	-10,438889
North	Rondônia (RO)	Vilhena	-60,145833	-12,740556
North	Acre (AC)	Rio Branco	-67,810000	-9,974722
North	Amazonas (AM)	Manaus	-60,025000	-3,101944
North	Roraima (RR)	Boa Vista	-60,673333	2,819722
North	Pará (PA)	Ananindeua	-48,372222	-1,365556
North	Pará (PA)	Belém	-48,504444	-1,455833
North	Pará (PA)	Benevides	-48,244722	-1,361389
North	Pará (PA)	Dom Elizeu	-47,505000	-4,285000
North	Pará (PA)	Marabá	-49,117778	-5,368611
North	Pará (PA)	Marituba	-48,341944	-1,355278
North	Pará (PA)	Rondon do Pará	-48,067222	-4,776111
North	Pará (PA)	Sta. Bárbara do Pará	-48,294444	-1,223611
North	Pará (PA)	Santarém	-54,708333	-2,443056
North	Pará (PA)	Tucuruí	-49,672500	-3,766111
North	Amapá (AP)	Macapá	-51,066389	0,038889
North	Tocantins (TO)	Araguaína	-48,207222	-7,191111
North	Tocantins (TO)	Palmas	-48,360278	-10,212778
Northeast	Maranhão (MA)	Bacabal	-44,791667	-4,291667
Northeast	Maranhão (MA)	São Luís	-44,302778	-2,529722
Northeast	Piauí (PI)	Parnaíba	-41,776667	-2,904722
Northeast	Piauí (PI)	Teresina	-42,801944	-5,089167
Northeast	Ceará (CE)	Caucaia	-38,653056	-3,736111
Northeast	Ceará (CE)	Fortaleza	-38,543056	-3,717222
Northeast	Ceará (CE)	Juazeiro do Norte	-39,315278	-7,213056
Northeast	Rio Grande do Norte (RN)	Caicó	-37,097778	-6,458333
Northeast	Rio Grande do Norte (RN)	Jardim do Seridó	-36,774444	-6,584444
Northeast	Rio Grande do Norte (RN)	Parnamirim	-35,262778	-5,915556
Northeast	Rio Grande do Norte (RN)	Mossoró	-37,344167	-5,187500
Northeast	Rio Grande do Norte (RN)	Natal	-35,209444	-5,795000
Northeast	Rio Grande do Norte (RN)	Pau dos Ferros	-38,204444	-6,109167
Northeast	Paraíba (PB)	Alagoa Grande	-35,630000	-7,158333
Northeast	Paraíba (PB)	Bayeux	-34,932222	-7,125000
Northeast	Paraíba (PB)	João Pessoa	-34,863056	-7,115000
Northeast	Paraíba (PB)	Santa Rita	-34,978056	-7,113889
Northeast	Paraíba (PB)	Souza	-38,228056	-6,759167
Northeast	Pernambuco (PE)	Araripina	-40,498333	-7,576111
Northeast	Pernambuco (PE)	Cabo de Sto Agostinho	-35,035000	-8,286667
Northeast	Pernambuco (PE)	Jaboatão dos Guararapes	-35,014722	-8,112778
Northeast	Pernambuco (PE)	Moreno	-35,092222	-8,118611
Northeast	Pernambuco (PE)	Olinda	-34,855278	-8,008889
Northeast	Pernambuco (PE)	Petrolina	-40,500833	-9,398611
Northeast	Pernambuco (PE)	Recife	-34,881111	-8,053889
Northeast	Pernambuco (PE)	Tamandaré	-35,104722	-8,759722
Northeast	Alagoas (AL)	Arapiraca	-36,661111	-9,752500
Northeast	Alagoas (AL)	Maceió	-35,735278	-9,665833
Northeast	Sergipe (SE)	Aracaju	-37,071667	-10,911111
Northeast	Sergipe (SE)	Barra dos Coqueiros	-37,038611	-10,908889
Northeast	Sergipe (SE)	Itabaiana	-37,425278	-10,685000
Northeast	Bahia (BA)	Barreiras	-44,990000	-12,152778
Northeast	Bahia (BA)	Eunápolis	-39,580278	-16,377500
Northeast	Bahia (BA)	Feira de Santana	-38,966667	-12,266667
Northeast	Bahia (BA)	Ilhéus	-39,049444	-14,788889
Northeast	Bahia (BA)	Itabuna	-39,280278	-14,785556
Northeast	Bahia (BA)	Jacobina	-40,518333	-11,180556
Northeast	Bahia (BA)	Jequié	-40,083611	-13,857500
Northeast	Bahia (BA)	Potiguará	-39,876667	-15,594722
Northeast	Bahia (BA)	Salvador	-38,510833	-12,971111
Northeast	Bahia (BA)	Teixeira de Freitas	-39,741944	-17,535000
Northeast	Bahia (BA)	Vitória da Conquista	-40,839444	-14,866111
Midwest	Mato Grosso do Sul (MS)	Campo Grande	-54,646389	-20,442778
Midwest	Mato Grosso do Sul (MS)	Corumbá	-57,653333	-19,009167
Midwest	Mato Grosso do Sul (MS)	Coxim	-54,760000	-18,506667
Midwest	Mato Grosso do Sul (MS)	Três Lagoas	-51,678333	-20,751111
Midwest	Mato Grosso do Sul (MS)	Ponta Porã	-55,725556	-22,536111
Midwest	Mato Grosso do Sul (MS)	Dourados	-54,805556	-22,221111
Midwest	Mato Grosso (MT)	Cuiabá	-56,096667	-15,596111
Midwest	Mato Grosso (MT)	Várzea Grande	-56,132500	-15,646667
Midwest	Goiás (GO)	Aparecida de Goiânia	-49,243889	-16,823333
Midwest	Goiás (GO)	Goiânia	-49,253889	-16,678611
Midwest	Goiás (GO)	Itumbiara	-49,215278	-18,419167
Midwest	Goiás (GO)	Luziânia	-47,950278	-16,252500
Midwest	Goiás (GO)	Novo Gama	-48,039444	-16,059167
Midwest	Goiás (GO)	Rio Verde	-50,928056	-17,798056
Midwest	Goiás (GO)	Uruaçu	-49,140833	-14,524722
Midwest	Distrito Federal (DF)	Brasília	-47,929722	-15,779722
Southeast	Minas Gerais (MG)	Belo Horizonte	-43,937778	-19,920833
Southeast	Minas Gerais (MG)	Formiga	-45,426389	-20,464444
Southeast	Minas Gerais (MG)	Januária	-44,361667	-15,488056
Southeast	Minas Gerais (MG)	Montes Claros	-43,861667	-16,735000
Southeast	Minas Gerais (MG)	Teófilo Otoni	-41,505278	-17,857500
Southeast	Minas Gerais (MG)	Ubá	-42,942778	-21,120000
Southeast	Minas Gerais (MG)	Uberaba	-47,931944	-19,748333
Southeast	Minas Gerais (MG)	Uberlândia	-48,277222	-18,918611
Southeast	Espírito Santo (ES)	Cach. de Itapemirim	-41,112778	-20,848889
Southeast	Espírito Santo (ES)	Cariacica	-40,420000	-20,263889
Southeast	Espírito Santo (ES)	Colatina	-40,630556	-19,539444
Southeast	Espírito Santo (ES)	Serra	-40,307778	-20,128611
Southeast	Espírito Santo (ES)	Viana	-40,496111	-20,390278
Southeast	Espírito Santo (ES)	Vila Velha	-40,292500	-20,329722
Southeast	Espírito Santo (ES)	Vitória	-40,337778	-20,319444
Southeast	Rio de Janeiro (RJ)	Cabo Frio	-42,018611	-22,879444
Southeast	Rio de Janeiro (RJ)	C. dos Goytacazes	-41,324444	-21,754167
Southeast	Rio de Janeiro (RJ)	Duque de Caxias	-43,311667	-22,785556
Southeast	Rio de Janeiro (RJ)	Itaperuna	-41,887778	-21,205000
Southeast	Rio de Janeiro (RJ)	Niterói	-43,103611	-22,883333
Southeast	Rio de Janeiro (RJ)	Nova Iguaçu	-43,451111	-22,759167
Southeast	Rio de Janeiro (RJ)	Rio de Janeiro	-43,207500	-22,902778
Southeast	Rio de Janeiro (RJ)	São Gonçalo	-43,053889	-22,826944
Southeast	Rio de Janeiro (RJ)	São João de Meriti	-43,372222	-22,803889
Southeast	Rio de Janeiro (RJ)	S. José do V. Rio Preto	-42,924444	-22,151389
Southeast	Rio de Janeiro (RJ)	Três Rios	-43,209167	-22,116667
Southeast	Rio de Janeiro (RJ)	Volta Redonda	-44,104167	-22,523056
Southeast	São Paulo (SP)	Araçatuba	-50,432778	-21,208889
Southeast	São Paulo (SP)	Barretos	-48,567778	-20,557222
Southeast	São Paulo (SP)	Bauru	-49,060556	-22,314722
Southeast	São Paulo (SP)	Botucatu	-48,445000	-22,885833
Southeast	São Paulo (SP)	Campinas	-47,060833	-22,905556
Southeast	São Paulo (SP)	Itapevi	-46,934167	-23,548889
Southeast	São Paulo (SP)	Itu	-47,299167	-23,264167
Southeast	São Paulo (SP)	Jandira	-46,902500	-23,527500
Southeast	São Paulo (SP)	Marília	-49,945833	-22,213889
Southeast	São Paulo (SP)	Presidente Prudente	-51,388889	-22,125556
Southeast	São Paulo (SP)	Ribeirão Preto	-47,810278	-21,177500
Southeast	São Paulo (SP)	Santana de Parnaíba	-46,917778	-23,444167
Southeast	São Paulo (SP)	Santos	-46,333611	-23,960833
Southeast	São Paulo (SP)	São Carlos	-47,890833	-22,017500
Southeast	São Paulo (SP)	São José do Rio Preto	-49,379444	-20,819722
Southeast	São Paulo (SP)	São Paulo (Pirituba)	-46,723611	-23,475000
Southeast	São Paulo (SP)	São Paulo (Ipiranga)	-46,642222	-23,543889
Southeast	São Paulo (SP)	São Sebastião	-45,409722	-23,760000
Southeast	São Paulo (SP)	Sorocaba	-47,458056	-23,501667
South	Paraná (PR)	Foz do Iguaçu	-54,588056	-25,547778
South	Paraná (PR)	Londrina	-51,162778	-23,310278
South	Paraná (PR)	Jacarezinho	-49,969444	-23,160556
South	Paraná (PR)	Maringá	-51,938611	-23,425278
South	Paraná (PR)	Palotina	-53,840000	-24,283889
South	Rio Grande do Sul (RS)	Crissiumal	-54,101111	-27,499722
South	Santa Catarina (SC)	Florianópolis	-48,549167	-27,596667
South	Santa Catarina (SC)	Itapiranga	-53,712222	-27,169444

*Diagnostic bioassays of temephos resistance* - The diagnostic bioassays were performed following the standardised procedures of the WHO (1981, 1992). The concentration of temephos required to identify resistant insects (i.e., the diagnostic concentration) was initially established as 14.0 µg a.i./L but was subjected to yearly calibration and validation with the standard susceptible Rockefeller strain, as described by Braga et al. (2004) and Macoris et al. (2005). The diagnostic concentration was applied as a 1 mL solution to each of the experimental containers, reaching a final 250 mL volume of contaminated water solution (except for the controls, for which only 1 mL acetone was used). Deionised and distilled water were used to prepare the bioassay solutions. Twenty-five individuals (3rd-4th instar mosquito larvae) were placed in 250 mL transparent glass containers containing temephos-contaminated water (except in the control treatments) and four replicates were used for each locally collected population. Mortality assessment of the mosquito larvae was performed after 24 h exposure. The larvae were considered dead if they were unable to rise to the surface when dorsally prodded.

*Geostatistical analyses* - These analyses were based on the geographical coordinates of each mosquito sampling site from which the mosquito populations were obtained and used to calculate the distance between sampling sites. The distances from the sampling sites and the mortality data obtained from the diagnostic bioassays were subjected to alternative kriging methods (stable, circular, spherical, exponential and Gaussian) to select suitable semivariogram functions for distance interpolation (Isaacs & Srivastava 1989). The semivariogram functions obtained using each group of models allowed the estimation of three parameters to determine their respective shapes: range (h_r_), partial sill (C), and nugget (C_o_). The range (h_r_) and partial sill (C) refer to the point in the semivariogram function in which a plateau is reached; the range (h_r_) corresponds to the distance at which this phenomenon takes place, while the partial sill (C) refers to its respective semivariance value. The nugget (C_o_) is the semivariogram value in which the model intercepts the *y*-axis (i.e., the mortality semivariance axis) corresponding to measurement errors or spatial sources of variation at distances smaller than the sampling interval (or both). Three additional parameters were calculated from these three basic parameters described above. These were: sill (C_o_ + C), proportion [C/(C_o_ + C)] and randomness (C_o_/C) of the data. A cross-validation procedure was subsequently used to select the best data adjustment to compare the observed and estimated data for each sampling point using the model of semivariogram function under test. This estimated error allows the best model selection as those leading to the error average closer to zero, aided by the randomness assessment (the higher, the better). The semivariance data obtained from the selected models were used to generate the spatial maps depicting the phenomenon of temephos resistance. All the spatial analyses were performed using ArcGIS 10 software (ESRI, Redlands, CA, USA).

## RESULTS

*General temephos mortality findings* - The diagnostic bioassays assessing mosquito larvae mortality by temephos were performed to estimate the frequency of temephos-resistant individuals in the sampled insect populations. This frequency of resistant individuals is indicated as an average mortality score ranging from 80.31% between 1999-2000 and dropping to less than 50% between 2010-2011 (Table II). The number of insect samples tested per year ranged from 25 (from 2010-2011) to 74 (between 2000-2001) and had a broad range of mortality response within each year, resulting in a high standard deviation of larval mortality per year (Table III).

**TABLE II:** Descriptive statistics of the diagnostic bioassays with temephos on larvae of the yellow fever mosquito *Aedes aegypti*

Year	Sampling sites (n)	Mortality (%)				Skewness (*g*_*1*_)	Kurtosis (*g*_*2*_)

		Minimum	Maximum	Mean	SD		
1999-2000	64	13.15	100.00	80.31	24.62	-1.22	3.40
2000-2001	74	10.80	100.00	71.53	26.34	-0.68	2.38
2002-2003	58	2.00	99.80	62.48	30.16	-0.51	2.08
2004-2005	59	1.50	98.45	53.41	33.69	-0.18	1.39
2006-2007	39	6.40	97.60	52.33	24.48	-0.16	1.97
2008-2009	46	6.00	96.70	50.60	24.99	0.05	1.82
2010-2011	25	7.50	88.20	49.99	28.16	-0.12	1.55

SD: standard deviation

**TABLE III:** Semivariogram models and parameters of larval mortality by temephos on populations of the yellow fever mosquito *Aedes aegypti*

Year	Kriging	Model	Nugget (C_0_)	Partial sill (C)	Sill (C_0_+C)	Proportion (C/C+C_0_)	Range (*h*_*r*_, m)	Randomness (C_0_/C)	Mean errors
1999-2000	Ordinary	Gaussian	132.963	639.079	772.042	0.827778	593820.368	0.208054	-0.027
2000-2001	Simple	Gaussian	231.740	640.182	871.922	0.734219	632424.376	0.361991	-0.059
2002-2003	Simple	Exponential	391.601	972.709	1364.31	0.712968	3658678.194	0.402588	-0.203
2004-2005	Ordinary	Gaussian	224.524	176.033	400.557	0.439471	695175.201	1.275465	0.101
2006-2007	Ordinary	Exponential	162.384	669.389	831.773	0.804774	1175553.465	0.242585	-0.096
2008-2009	Ordinary	Circular	57.218	723.989	781.207	0.926757	947927.124	0.079032	0.266
2010-2011	Ordinary	Circular	367.832	262.731	630.563	0.416661	507101.080	0.714269	1.576

**Fig. 2:**
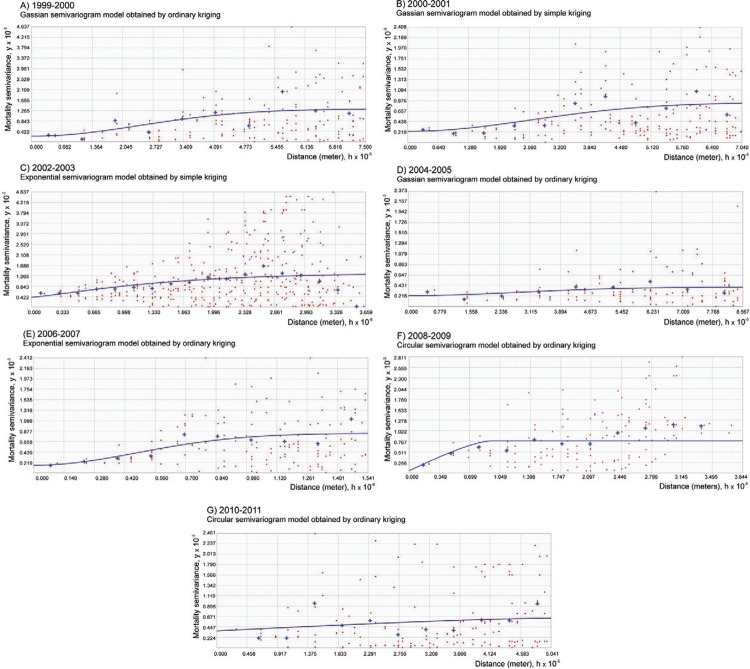
: semivariogram models [mortality semivariance (y) as a function of distance (x)] exhibited in Table II and obtained from the diagnostic bioassays of temephos resistance on larvae of the yellow fever mosquito *Aedes aegypti.* Observed points are represented as red symbols, and averages are represented as blue crosses.

*Semivariogram model selection* - Suitable semivariogram models were obtained for each biannual dataset of temephos mortality using the diagnostic insecticide resistance bioassays. The selected semivariogram models are exhibited in Table III, along with their respective parameters for model selection. The plots from each model and the respective observed data are exhibited in Fig. 2.

**Fig. 3:**
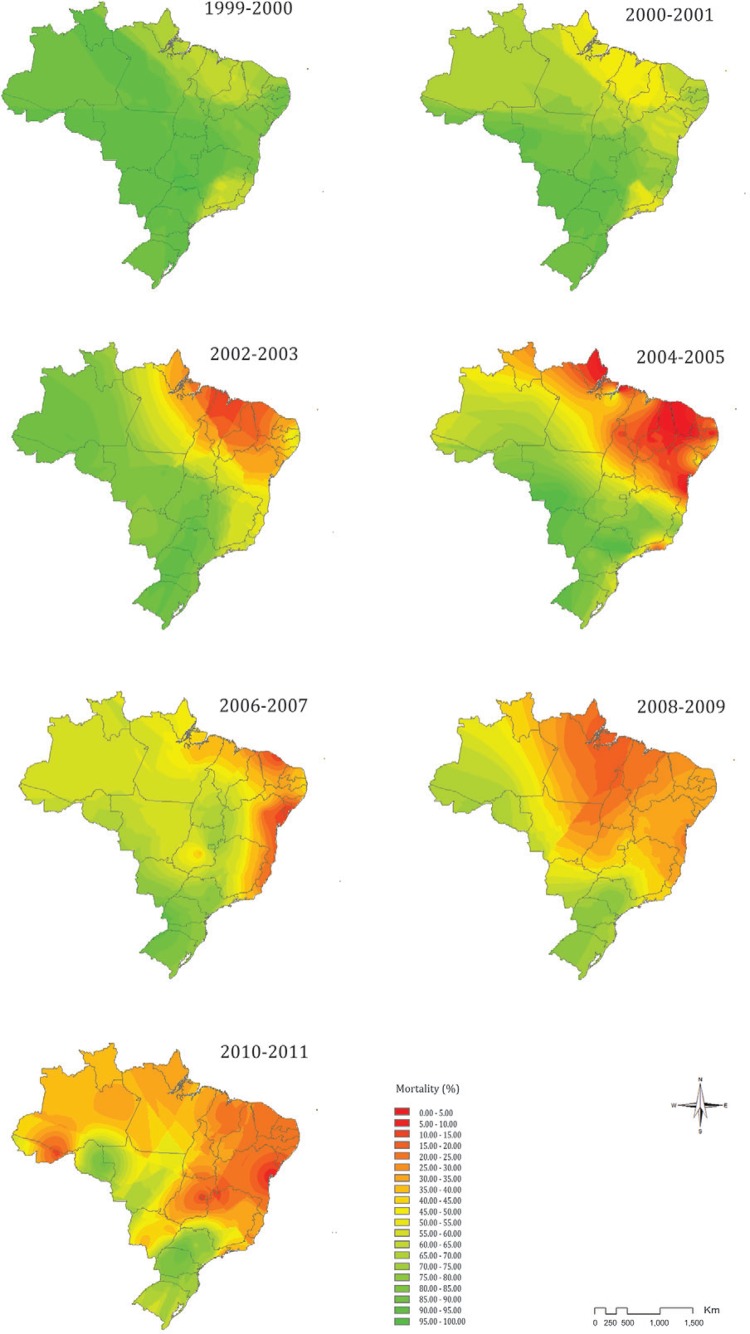
: contour maps of temephos resistance in Brazilian populations of the yellow fever mosquito (*Aedes aegypti*) generated using spatial interpolation. The colour legend indicates the represented range of mortality (%) of mosquito larvae obtained in the temephos resistance diagnostic bioassays. Colours tending toward red indicate lower larval mortality and, consequently, a higher frequency of temephos resistance.

*Temporal spread of temephos resistance* - Spatial interpolation using kriging allowed mapping the country-wide spread of temephos resistance in larvae of YF mosquitoes from 1999-2000 until 2010-2011, which is the last year the survey data were available. Initially the efficacy of temephos was high, causing larval mortality of > 80% throughout Brazil, except in the coastal area, which spans from Pará in the north to Piauí in the northeast and encompasses the state of Rio de Janeiro and neighboring parts of São Paulo and Minas Gerais (Fig. 3). However, the frequency of temephos resistant individuals in the insect populations increased steadily during each biannual survey, reflecting a significant reduction in temephos efficacy. This trend reached high levels (< 50% mortality) in about half the country as early as 2004-2005 (Fig. 3). Although the frequency of temephos resistance seems to have been attenuated in the main problem areas observed between 2004-2005, temephos resistance continued to spread within Brazil. By 2010-2011 only Rondônia (in the North), São Paulo (Southeast), Paraná and Santa Catarina (South) exhibited satisfactory temephos efficacy against YF mosquito larvae. New focal areas of temephos resistance were detected in the 2010-2011 survey radiating from near Rio Branco (southern Acre in North Brazil, near Bolivia) and Brasilia (Central Brazil), leading to a country-wide resistance phenomenon.

## DISCUSSION

The temephos mortality dataset obtained from the diagnostic bioassays performed by the MoReNAa, although not carried out with the objective of spatial interpolation to generate temephos resistance maps for Brazilian populations of *Ae. aegypti*, allowed such interpolations and the inferences necessary to generate the maps. The effort provided a means to clearly illustrate the temporal spread and spatial reach of temephos resistance in *Ae. aegypti* - which greatly increased during the 12-year period of assessment - within the Brazilian territory. Nonetheless, a more fine-tuned survey focusing on diagnostic bioassays of insecticide resistance using larger and better-distributed sampling sites would allow even more comprehensive assessments for eventual decision-making regarding policies and procedures to be adopted.

Temephos resistance among Brazilian populations of the YF mosquito is far from novel. All the Brazilian states have adopted the routine use of temephos (1% sand granule formulations) to manage *Ae. aegypti* by controlling its larvae since the early 1990’s (Funasa 1994, 2001, Sucen 1997). The result of this continuous and consistent use of temephos throughout the country led to reports of temephos resistance as early as 1995 (Macoris et al. 1995). The increased incidence of dengue during the 1990s in Brazil attributed to the spread of *Ae. aegypti* enhanced concern regarding insecticide use against the mosquito and the susceptibility of mosquito populations (da Silva Jr et al. 2002, Braga & Valle 2007). The end result was the establishment of an insecticide-resistance monitoring program in the country that focused on populations of the YF mosquito (Braga & Valle 2007). Consistent detection of temephos resistance in different parts of the country soon followed (Campos & Andrade 2003, Lima et al. 2003, 2006, Macoris et al. 2003, 2007, Melo-Santos et al. 2010, Gambarra et al. 2013, Diniz et al. 2014).

Some of the studies of temephos resistance among Brazilian populations of the YF mosquito explored the mechanisms involved and the existence of fitness costs associated with this resistance. Fitness costs were indeed detected (Diniz et al. 2014). Unfortunately, the studies on the underlying mechanisms of temephos resistance were more confused and patchy, but they were suggestive of the prevailing involvement of enhanced insecticide detoxification as the main mechanism, with esterases likely playing a major role, although not an exclusive one (Braga & Valle 2007, Melo-Santos et al. 2010, Gambarra et al. 2013, Macoris et al. 2014). These findings seem consistent with mechanistic studies of temephos resistance performed with other Latin American populations of the same species (Bisset Lazcano et al. 2009, Bisset et al. 2013, Grisales et al. 2013, Reyes-Solis et al. 2014, Saavedra-Rodriguez et al. 2014).

The twelve-year effort of the MoReNAa achieved a great deal, but no summary of the country-wide survey effort had ever been performed; therefore, creating such a summary was the objective of the current work. The geostatistical tools used here allowed the recognition of both the temporal pattern of the spread of temephos resistance in the country and its gravity by 2011. Despite the early detection of temephos resistance in the mid-1990s, country-wide use of temephos continued; consequently, temephos resistance spread throughout the country during the following years, reaching serious levels by 2002-2003. At this point, nearly half of the country was already having problems because of temephos resistance in *Ae. aegypti*, particularly when considering “resistant” to mean mosquito populations that exhibit mortality levels below the 80% threshold-a threshold that incurs in a high likelihood of control failure (Davidson & Zahar 1973). The scenario has simply gotten worse in subsequent years. Now, nearly all the country (except a part in the South) exhibits temephos resistance.

The use of temephos as a mosquito larvicide in Brazil has been suppressed since late in 2010, which may reverse the spread of resistance and allow for future use of the compound. However, the high frequency of resistant individuals already established in the country potentially limits the extent of such (future) use, even if the fitness cost associated with temephos resistance prevails in the country. Effective, safe and cheap insecticides such as temephos, which are the underlying reasons for its global use as a mosquito larvicide, are hard to come by (Tomé et al. 2014). A few alternatives have emerged and are currently being explored, including a few pyrethroids, but these already exhibit insecticide resistance problems in wide areas in Brazil (e.g., Brito et al. 2013). More recently, insect growth regulators and the bioinsecticide *Bacillus thuringiensis* serovar *israelensis* (Bti) have been explored (Braga & Valle 2007, Fontoura et al. 2012, Araújo et al. 2013).

In conclusion, temephos resistance in Brazilian populations of the YF mosquito spread during the 12-year survey period, showing that resistance is now widespread and there is little hope of achieving effective mosquito control with this insecticide. Alternative insecticides aided by the preventive elimination of potential mosquito breeding sites are necessary. However, the use of these alternative insecticides will also lead to the eventual emergence of resistant mosquito populations - and may have already occurred in the country, considering their present rate of use. Therefore, continuing country-wide surveys are necessary to guide management decisions by the national health officers. In such a context, planned yearly systematic sampling and insecticide resistance diagnostic bioassays are necessary. Moreover, geostatistical analyses to map the levels and spread of the phenomenon are also necessary.
